# Electroresistance in multipolar antiferroelectric Cu_2_Se semiconductor

**DOI:** 10.1038/s41467-021-27531-x

**Published:** 2021-12-10

**Authors:** Hui Bai, Jinsong Wu, Xianli Su, Haoyang Peng, Zhi Li, Dongwang Yang, Qingjie Zhang, Ctirad Uher, Xinfeng Tang

**Affiliations:** 1 State Key Laboratory of Advanced Technology for Materials Synthesis and Processing, Wuhan University of Technology, 430070 Wuhan, China; 2 Nanostructure Research Center, Wuhan University of Technology, 430070 Wuhan, China; 3 Department of Physics, University of Michigan, Ann Arbor, MI 48109 USA

**Keywords:** Ferroelectrics and multiferroics, Thermoelectrics

## Abstract

Electric field-induced changes in the electrical resistance of a material are considered essential and enabling processes for future efficient large-scale computations. However, the underlying physical mechanisms of electroresistance are currently remain largely unknown. Herein, an electrically reversible resistance change has been observed in the thermoelectric *α*-Cu_2_Se. The spontaneous electric dipoles formed by Cu^+^ ions displaced from their positions at the centers of Se-tetrahedrons in the ordered *α*-Cu_2_Se phase are examined, and *α*-Cu_2_Se phase is identified to be a multipolar antiferroelectric semiconductor. When exposed to the applied voltage, a reversible switching of crystalline domains aligned parallel to the polar axis results in an observed reversible resistance change. The study expands on opportunities for semiconductors with localized polar symmetry as the hardware basis for future computational architectures.

## Abstract (web summary)

The reaction of a conductive ferroelectric matter to external electric field remains largely unknown. Here, the authors reveal the relationship between the electrically-driven crystalline domain transition along the multiple-polar directions and the resistance change.

## Introduction

Electroresistance is a phenomenon whereby the resistance of a substance can be electrically tuned by an application of electric field^1–3^. Reversible alterations (switching) of the resistance in electronic devices called memristors is viewed as the essential effect for the development of future computing architectures and energy efficient large-scale computing^4–6^. Several mechanisms underpinning the electroresistance have been proposed, among them the electrochemical growth and dissolution of metallic filaments^6–10^, repeated formation and rupture of the electric conduction paths in insulators^11^, and voltage-controlled domain configurations in ferroelectric tunnel barriers^2,12,13^. Nevertheless, given the complexity of the materials involved, the underlying mechanisms remain largely unclear. In a metal-insulator-metal (MIM) memory cell, for instance, the insulator layer can be constructed by a wide range of binary and multinary oxides^14^, chalcogenides^15^, as well as organic compounds^16^. The lack of practical methods of directly observing the microstructural evolutions related to the resistance change has long been a roadblock in clearly resolving the underlying physical mechanism. With the recent development of in-situ transmission electron microscopy (TEM)^17–19^, microstructural evolutions can be monitored by both imaging and diffraction, while the applied electric fields/currents are applied and measured. This provides a convenient and practical way to investigate the causes of resistance changes at atomic resolution.

Although as early as in 1965, Anderson and Blount predicted the existence of “ferroelectric metals”, where a ferroelectric-like structural transition was supposed to occur in the metallic state^20,21^, the screening effect of free charges would make the net electric field inside metals/semiconductors zero, according to Gauss’s law^21^. Unlike traditional insulating ferroelectrics, conductive and ferroelectric metals/semimetals were not proved experimentally until recently^22–24^. Ferroelectrics are normally characterized by the presence of polar point groups that exhibit polarity along the polar axes^24^. However, there are a vast number of oxides and chalcogenides, which have nonpolar point groups allowing for the presence of multiple local polar axes. Among them, the coexistence of conductivity (or semi-conductivity) along with topological multi-polarity is highly possible. Moreover, the multipolar ordered phases may possess fascinating electronic and dynamic atomic structures under the applied stimuli enabling unprecedented functionalities. *α*-Cu_2_Se is a *p*-type semiconductor (the charge carriers being positively charged holes) with quite a small bandgap of ≈0.84 eV^25^ that has been intensively studied as a thermoelectric material due to its unique physical properties^26,27^. In the structure, Se-anions constitute a rigid cubic sub-lattice while Cu cations form a so-called “liquid-like” sub-lattice, a reflection of the complexity and difficulties in the precise determination of the Cu-ion positions^28–31^. The complex domain structure in *α*-Cu_2_Se makes it difficult to solve uniquely the atomic structure of the room-temperature phase by x-ray diffraction^32,33^.

In this work, the crystalline structure is studied by electron diffraction having the advantage of resolving small size crystalline domains. Meanwhile, we apply in-situ TEM to investigate the dynamic microstructural evolution under the influence of an externally applied voltage or electric current. Based on the atomic structure, the Cu^+^ ion positions are carefully analyzed with the emphasis on their deviations from the central position of the Se-tetrahedra (the characteristic of a ferroelectric material). It is revealed that *α*-Cu_2_Se has several localized “antiferroelectric-like” dipoles, oriented along the three principal axes. The material can thus be termed a multipole antiferroelectric. We then focus on establishing a relationship between the electrically driven crystalline domain reorientation/transition along the multiple polar directions and the resistance change, the underlying mechanism of electroresistance in thin *α*-Cu_2_Se crystals.

## Results and discussion

### Electroresistance correlated with reversible switching of crystalline domains

**Fig. 1:**
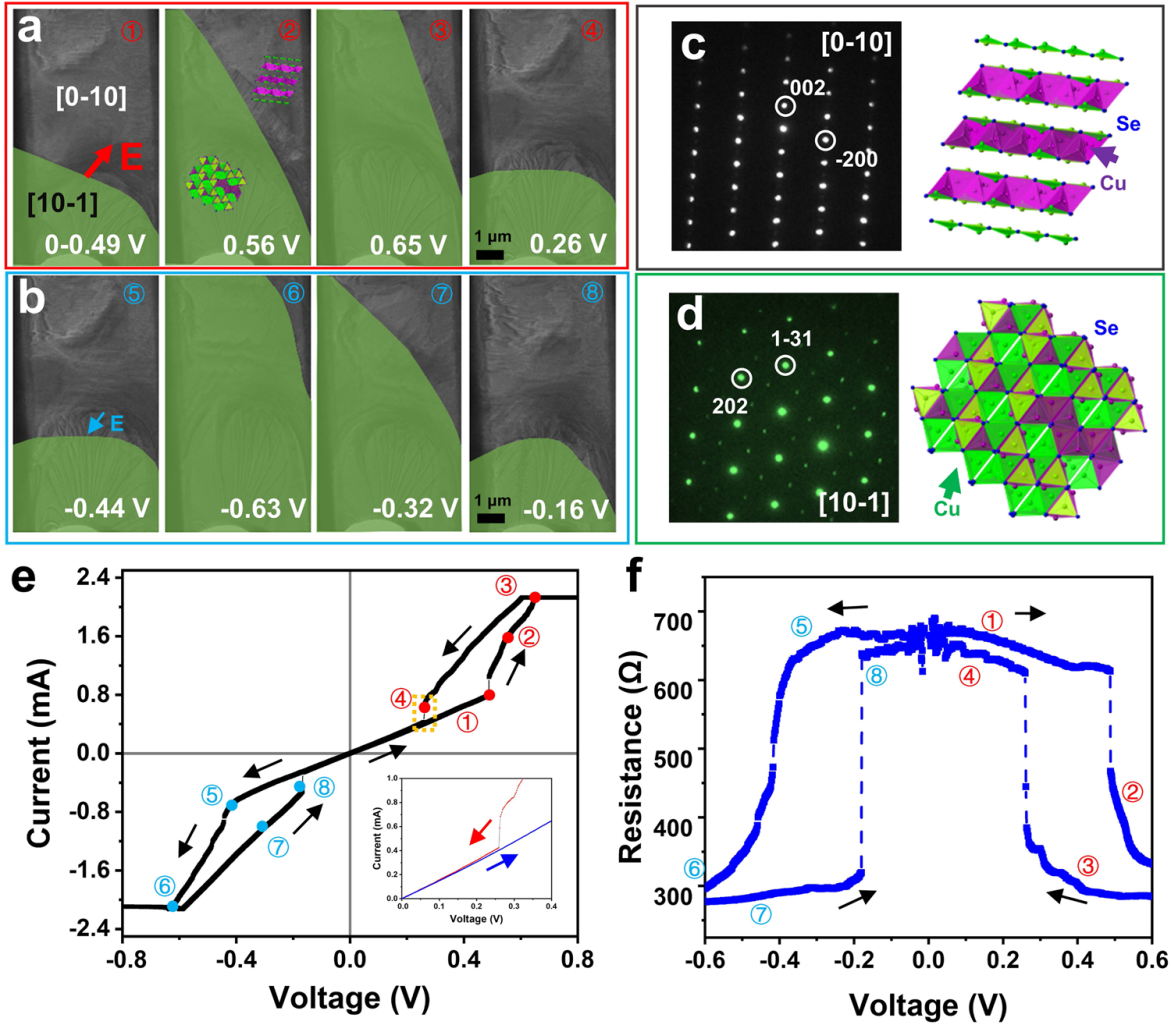
Electroresistance of *α*-Cu_2_Se correlated with reversible switching of crystalline domains. **a** TEM images of the boundary between the two domains (the green area marks the [10-1] domain) during the positive bias of 0–0.49 V, 0.56 V, 0.65 V, and 0.26 V, respectively. **b** TEM images of the boundary between the two domains during the negative bias of −0.44 V, −0.63 V, −0.32 V and −0.16 V, respectively. **c**, **d** the corresponding selected area electron diffraction patterns of *α*-Cu_2_Se along the [0-10] and [10-1] directions, and illustrated atomic models of the two domains, respectively. **e** Current–voltage characteristics of Cu_2_Se under the bias voltage. The insets show the enlarged view in the yellow dashed box. **f** Resistive-voltage characteristics change of *α*-Cu_2_Se during the voltage cycle of 0-0.6-0-(−0.6)-0 V.

**Fig. 2:**
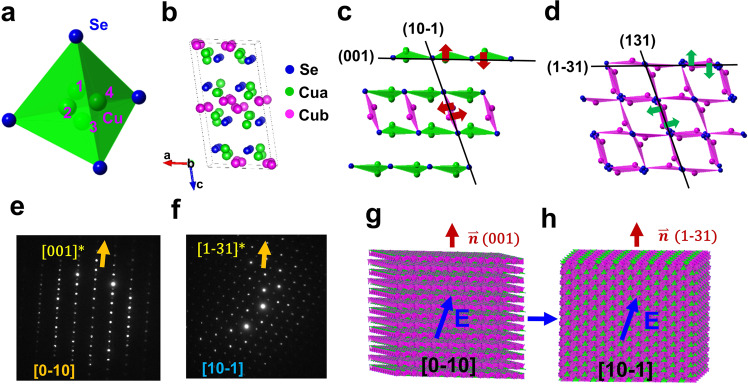
The multipole antiferroelectric axis identified in α-Cu_2_Se and the voltage-driven, topological domain reorientation. **a** Illustration of the Cu–Se tetrahedron in disordered *β*-Cu_2_Se showing four equivalent occupation sites. **b** Crystal structure of α-Cu_2_Se, blue: Se, green: Cu1, purple: Cu2. **c**, **d** Illustration of the Cu-Se tetrahedron in ordered *α*-Cu_2_Se, showing the localized dipoles formed in the structure along different directions. The locally polarized direction along the normal vector of (001) and (10-1) are shown by red arrows, and that along the normal vector of (131) and (1-31) by green arrows. **e**, **f** SAED patterns disclosing the orientation relationship between the two domains tuned by the applied voltage: [0-10]//[10-1], (001)//(1-31) and (10-1)//(131). **g**, **h** Illustration of the atomic structure of the two domains whose orientation can be tuned by the electric field.

The electrically driven domain reorientation in thin *α*-Cu_2_Se crystals corresponding to resistance changes has been observed with the aid of in-situ TEM (Supplementary Fig. 1) under various voltages applied to the crystals and the corresponding electric currents measured. Although there are many structural variants^31–34^, the *α*-Cu_2_Se sample used in our experiments is determined to have the monoclinic structure (*a* = 7.148 Å, *b* = 12.349 Å, *c* = 13.833 Å, *β* = 100°)^33^. As shown in Fig. 1a, before the external voltage was applied, the thin *α*-Cu_2_Se sample had two domains, namely the [10-1] and [0-10] domains (determined by the electron diffraction patterns shown in Fig. 2c, d, respectively), with a curved boundary. When the applied voltage was increased from 0 to 0.49 V, the boundary between the two domains remained almost unchanged, while the resistance was about ~650 Ω (Fig. 1e, f). When the applied voltage reached above 0.49 V, a sudden migration of the domain boundary toward the electric-field direction was observed (Fig. 1a and Video S1). The migration continued with the further increase of the voltage. As the domain migration commenced, a small resistance state (about ~300 Ω) appeared (the measured current reached the compliance current after 0.6 V). During the downside cycling, when the voltage was decreased to ~0.26 V, a portion of the [10-1] domain transformed back into the [0-10] domain (Fig. 1a), while the resistance switched back to a high state (about ~650 Ω), as shown in Fig. 1e, f. The domain configuration and the corresponding resistance did not return to their exact original state, showing some hysteresis (similar to that in a synapse where the previous effect was partially recorded)^35,36^.

When direction of the applied voltage was changed, a similar phenomenon was observed (Fig. 1b). The change in the electroresistance of thin *α*-Cu_2_Se with voltage-cycling is highly repeatable (Supplementary Figs. 2–5 and Videos S2–S4) and electrically unipolar (or symmetric), meaning that the process of transformation does not depend on the polarity of the applied voltage, as shown in Fig. 1 and Supplementary Fig. 9. At a high applied voltage (either positive or negative), the portion of the [10-1] domain increased corresponding to a decrease of the resistance. When the voltage was cycled back to zero, the high resistance state was substantially recovered while there remained some hysteresis in the domain configuration and resistance.

### *α*-Cu_2_Se is a multipole antiferroelectric semiconductor

From the structural deviation of the cations’ (Cu^+^) centers from those of anions (Se^2−^), the semiconducting *α*-Cu_2_Se can be defined as having localized antiferroelectric arrangements of multipolar order. As shown in Fig. 2a, at high temperatures, the cubic *β*-Cu_2_Se nonpolar high‐symmetry phase is characterized by the Cu^+^ ions occupying any of the four possible Cu sites in a disordered fashion, viewed equivalently as sitting at the center of the Se-tetrahedron. The overlap of the centers of positive and negative ions makes the high‐symmetry phase non-ferroelectric. Indeed, the temperature where the phase transition from the ordered *α*-Cu_2_Se phase to the disordered *β*-Cu_2_Se phase (*fcc*, space group ${Fm}\bar{3}m$, *a* = 5.84 Å) takes place is alike to the Curie temperature in a typical ferroelectric crystal. However, the occupation of Cu^+^ ion sites in *α*-Cu_2_Se is ordered, as shown in Fig. 2b. For clarity (to show the polarity), only the triangle (the closest to the Cu^+^ ion) in the Cu-Se tetrahedron (as illustrated in Supplementary Fig. 6) is shown in Fig. 2c, d. In the illustration, the locally polarized directions corresponding to the deviation vectors of positive and negative ions are shown by red arrows (along the normal vector of (001) and (10-1)), and green arrows (along the normal vector of (131) and (1-31)), respectively. Pairs of oppositely oriented dipoles are identified and are shown by the arrows (Fig. 2c, d and Supplementary Fig. 7). Similar to conventional antiferroelectric dipoles, the polarity of the localized dipole pair is opposite so that the crystal is electrically neutral. Unlike the traditional antiferroelectric structure, several dipolar directions are identified in the structure, namely along the normal vectors of (001), (10-1), (131), and (1-31), the feature termed as “multipole ferroelectric”.

### Topological domain transition among the multiple-polar axes

Under the applied electric field, the crystalline orientation of *α*-Cu_2_Se can be switched among the available multipolar directions, similar to a topological transition that leads to a quantized orientation of domain transformations. Here, the quantized transition refers to the limited number of possible transitional orientations defined by the dipolar directions. Under the applied voltage, we have experimentally observed domain transitions from the [0-10] domain to the [10-1] domain, as revealed by electron diffraction (Fig. 2e, f and Supplementary Fig. 8). In the transition, the normal vector of the (001) plane (before) is parallel to the normal vector of the (1-31) plane (after) and both of them become the <111> direction if the crystalline symmetry is treated as a pseudocubic cell (or inherited from the pseudocubic <111> axis in the high temperature, cubic phase). The observed transition can thus be considered as a geometrically topological transition among several well-defined crystalline orientations induced by the applied electric field/voltage, as illustrated in Fig. 2g, h and Supplementary Fig. 8.

### Atomic mechanism of the voltage-driven, topological domain reorientation

**Fig. 3:**
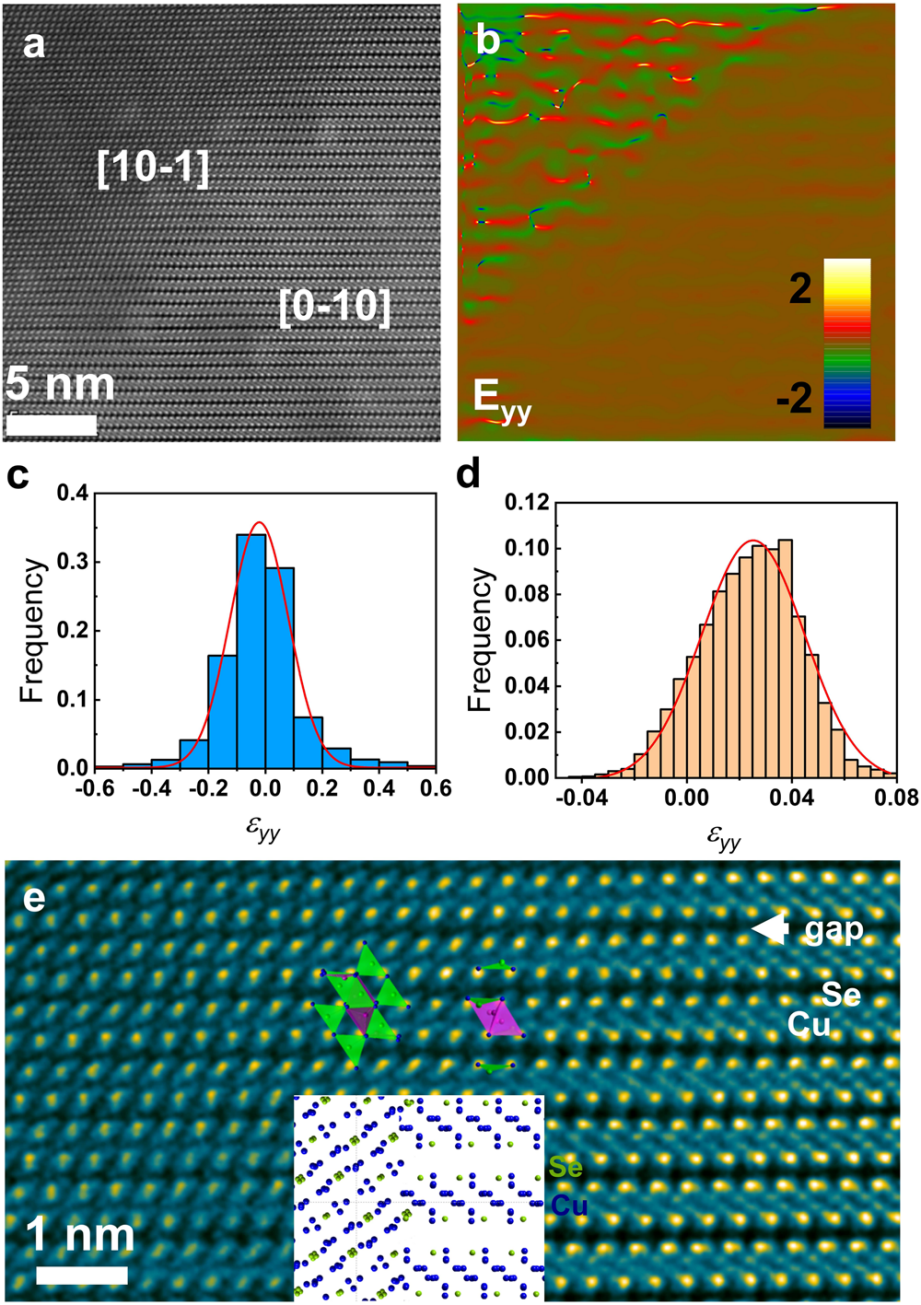
Atomic structure and strain distribution at the domain interface. **a** Atomic resolution HAADF-STEM image of the domain interface, showing clearly the layer-to-layer gaps in the [0-10] domain. **b** The strain map along the yy direction of the domain interface calculated by GPA based on **a**. **c**, **d** The strain distribution within the [10-1] domain and the [0-10] domain, respectively. **e** Atomic images of the domain interface at high magnification. The insets show atomic model of the interface.

The electrically driven *α*-Cu_2_Se domain transition among the multiple polar axes is realized by a collective hopping of Cu^+^ ions, while the Se^2−^ lattice remains almost intact (but subjected to elastic strain due to the transition). The atomic structure of the domain interface has been studied by high-angle annular dark field (HAADF) scanning transmission electron microscopy (STEM), as shown in Fig. 3a and Supplementary Fig. 9. Se^2−^ ions have a brighter contrast (due to their larger atomic number) than Cu^+^ ions and the Se^2−^ lattice is continuous and coherent across the interface. However, a large elastic strain is found in the [10-1] crystalline domain (20% compared to ~0.4% in the [0-10] domain), as shown in Fig. 3b and Supplementary Fig. 10. Unlike the Se^2−^ ions, the Cu^+^ ions undergo a significant rearrangement, i.e., the layer-to-layer gaps (as illustrated in Fig. 3e and Supplementary Fig. 11) have been filled when the [0-10] domain transformed into the [10-1] domain. Atomically, a collective hopping of the Cu^+^ ions takes place as illustrated in the inset of Fig. 3e and Supplementary Fig. 11, where, for instance, the Cu2 ion hops to fill the gap. Such collective hopping is not random (nothing like a liquid behavior) and it leads to a swift change in the crystalline orientation (domain transition). A clear migration of domain boundaries with the applied voltage is observed in the high-resolution transmission electron microscopy (HRTEM), which demonstrates the collective immigration of Cu^+^ ions (Supplementary Fig. S12 and Video S5).

Since the *α*-Cu_2_Se structure is anisotropic (i.e., the parallel plane conductivity is higher than the conductivity normal to the plane, the electrical conductivity along different directions of *α*-Cu_2_Se calculated by density functional theory (DFT), as shown in Supplementary Fig. 13, the electrical resistance will be altered once the geometrical arrangement between the applied electric field and the crystalline orientation is changed. Due to the layered structure of Cu_2_Se and the gaps between the layers, the anisotropic behavior of the *α*-Cu_2_Se structure is exemplified by its electrical conductivity. It explains why more [10-1]-type domains were present in thin Cu_2_Se crystals, and thus the higher electrical conductivity was observed in the experiment. Among the three polar directions, the <001> axis points along the plane normal of the Cu_2_Se layer, which has the lowest electrical conductivity. The other two polar axes are within the Cu_2_Se layer and have a relatively high electrical conductivity.

Unlike the conventional insulating antiferroelectric, the conducting antiferroelectric has a different response to the applied electric field. Under a static electric field (in the absence of an electrical current), the external electric field will be screened out in a conductor, thus little effect will be observed in the conducting antiferroelectric. Once it is connected and an electric current passes through, the localized antiferroelectric-type structure will have an additional resistance effect and an extra voltage-drop will develop. Once the local voltage reaches the threshold, collective hopping of Cu^+^ ions happens, leading to the crystalline domain transition along the multiple polar directions and the resistance change, which is the underlying mechanism of electroresistance in thin α-Cu_2_Se crystals. The effect can also be described as a voltage-driven, topological and quantized crystalline domain reorientation. As an ion-conductor, *α*-Cu_2_Se has a quite low threshold voltage to trigger the hopping of Cu^+^ ions^37^. A nudged elastic band (NEB) method^38^ is used to simulate the cross-layer migration of Cu^+^ ions. As Supplementary Fig. S14 shows, the migration barrier of the Cu^+^ ions is quite low 0.114 and 1.4 eV). In the experiment, it is observed that when the positive voltage is reduced below the threshold (say to 0.4 V), the Cu^+^ ions hop back to their original positions (leading to the domain reorientation and increase of the resistance). The reversibility under the same polarity voltage (either positive or negative) demonstrates that long-range diffusion of Cu^+^ ions (and chemical reduction of Cu^+^ to neutral Cu) can be mostly ignored.

### Strain-induced switching of domains in the antiferroelectric semiconductor

The reversed domain transition from a low-resistance state back to a high-resistance state is driven mainly by mechanical strains (induced due to the domain reconfiguration). From the rapid change of the contrast in TEM images (Video S1), it is clear that the strain field has been established by the applied voltage in the thin Cu_2_Se crystal. A strain map of the interface of the two domains calculated by the method of geometric phase analysis (GPA)^39^ is shown in Fig. 3b and Supplementary Fig. 11, in which the strains (mainly due to the Se sub-lattice) is almost relaxed in the [0-10] domain by the layer-to-layer gaps. However, the unevenly distributed strain field is clearly seen in the [10-1] domain. Both the amplitude and frequency of the strain in the [10-1] domain are much larger than the one measured in the [0-10] domain (Fig. 3c, d). Once the voltage is reduced below the threshold, the relaxation of the strain within the [10-1] domain (induced by the applied voltage) leads to the formation of the [0-10] domain with the layer-to-layer gaps. In other words, due to the mechanical strain, the Cu^+^ ions collectively move back to their original sites once the voltage is below the threshold, the effect similar to the piezoelectricity (where a ferroelectric/antiferroelectric material has intrinsically a piezoelectric property).

### Hysteresis in the resistance and domain configuration

During the voltage cycle, there is a hysteresis in the resistance and domain configuration, as well as the strain field within the domain. After several cycles, residual strains similar to those generated by the gaps in the Cu_2_Se layers in the [0-10] domain were found in the [10-1] domain, as shown in Supplementary Fig. 15. The layered distributed strains are clearly seen in the GPA-processed images (Supplementary Fig. 15c–e). As shown in Fig. 3b, the mechanical strain evolves hand in hand with the reversible domain transition. As already noted, the strain induced by the applied high voltage is not completely relaxed when the voltage is turned off and the hysteresis emerges.

In summary, *α*-Cu_2_Se is an “antiferroelectric” semiconductor with multiple local polar axes that shows a symmetric electroresistance effect under voltage cycling. The resistance change is caused by the electric or strain-induced switching of crystalline domains that have different electrical conductivity. Atomically, the collective hopping of Cu^+^ ions triggered by electric fields or mechanical strains is the cause of the quantized orientation transformation of domains along the well-defined local polar axes, an intriguing phenomenon observed in conductive antiferroelectric semiconductors. The present work establishes a relationship between the electrically-driven crystalline domain transition along the multiple polar directions and the resistance change, the underlying mechanism of electroresistance in thin *α*-Cu_2_Se crystals. The effect can also be described as a voltage-driven, topological and quantized crystalline domain reorientation.

## Methods

### Materials synthesis

Single crystals with nominal composition of Cu_2_Se were synthesized via melting-annealing processes. High purity raw elements Cu (99.99%) and Se (99.99%) were weighed out according to the stoichiometric ratio of 2:1 and enclosed in a fused silica tube. The raw material was melted at 1423 K for 12 h, and then slowly cooled down to 873 K in 100 h. After the reaction temperature was kept constant for 100 h, the tubes were naturally cooled to room temperature. The obtained ingots consisted solely of *α*-Cu_2_Se. The ingots were crushed into small pieces, and small single crystals were manually selected in air.

### Instrumentation

The selected small single crystals were shaped and reduced in size by focused ion beam (FIB) milling (Helios Nanolab G3 UC, FEI) for the structural characterization and in-situ TEM observation. in-situ TEM characterization for structural evolution was carried out by transmission electron microscopy (Talos F200s, FEI) and a double-tilt TEM-STM electrical holder provided by ZEPTools Technology Company. The atomic resolution HAADF-STEM images were obtained by a double C_S_-corrected transmission electron microscopy (Titan Themis G2 60–300, FEI). A continuous series of drift-corrected images were averaged to reduce scanning noise and sample drift during the acquisition process.

### Computational details

We performed the density functional theory (DFT) calculations by using Vienna Ab initio Simulation Packages (VASP) implemented with projector-augmented wave (PAW) method. The generalized gradient approximation (GGA) by Perdew, Burke, and Ernzerhof (PBE) was chosen as the exchange-correlation functional. We set the plane-wave cutoff energy at 500 eV. The experimental crystal structure data were adopted as the initial structure, and then relaxed with the energy and force convergence criteria of 10^−8^ eV and 0.01 eV Å^−1^. Eigenvalues of all the electronic states in the relaxed structure were then sampled with a dense Gamma-centered *k*-mesh with the spacing of 0.01 Å^−1^ between every two *k*-points, which were used in calculations of the electrical conductivity *σ*. The electrical conductivity divided by relaxation time *σ*/*τ* was calculated by solving the linearized Boltzmann transport equation (BTE) based on the constant relaxation time (CRT) approximation, which has been encoded in BoltzTraP2. The evaluation of *τ* is nontrivial. Given that *τ* is generally considered direction independent even in some highly anisotropic structures, we used *σ*/*τ* versus carrier concentration along three lattice vectors to give the qualitative conclusion on the anisotropy of electrical conductivity without considering the influence from *τ*. An energy difference of 1.0 × 10^−8^ eV/atom was set to obtain accurate electronic ground-state calculation. The maximum force tolerance was set to 0.01 eV/Å for structural optimization. The k-point mesh utilized was up to (5 × 3 × 2) in the gamma centered Monkhorst-Pack Grid using vaspkit packages. The DFT-D3 method with Becke-Jonson damping was used to correct the dispersion force. The climbing image nudged elastic band (CI-NEB) method was carried out for the diffusion simulation of the Cu atom in the Cu_2_Se systems, with three climbing images between initial and final states generated through the transition state tools for VASP (VASP + VTST).

## Supplementary information

Supplementary Information

Supplementary Movie 1

Supplementary Movie 2

Supplementary Movie 3

Supplementary Movie 4

Supplementary Movie 5

## Data Availability

All data are available in the main Article and Supplementary Information, or from the corresponding author upon reasonable request.
