# Supplementary material for: Electroresistance in multipolar antiferroelectric Cu2Se semiconductor
Source: Nat Commun. 2021 Dec 10;12:7207. doi: 10.1038/s41467-021-27531-x (PMC8664818; doi:10.1038/s41467-021-27531-x)
Supplement: Supplementary file 1 — Supplementary Information [file 41467_2021_27531_MOESM1_ESM.pdf]

**Supplementary Information for**

# Electroresistance in multipolar antiferroelectric $Cu_2Se$ semiconductor

Hui Bai[1,2†], Jinsong Wu[1,2†*], Xianli Su[1*], Haoyang Peng[1,2], Zhi Li[1], Dongwang Yang[1],

Qingjie Zhang[1], Ctirad Uher[3] and Xinfeng Tang[1*]

[1]*State Key Laboratory of Advanced Technology for Materials Synthesis and Processing, Wuhan University of Technology, Wuhan 430070, China*

[2]*Nanostructure Research Center, Wuhan University of Technology, Wuhan 430070, China*

[3]*Department of Physics, University of Michigan, Ann Arbor, Michigan 48109, USA*

†These authors contributed equally to this work.
*Correspondence and requests for materials should be addressed to Jinsong Wu (wujs@whut.edu.cn), Xianli Su (suxianli@whut.edu.cn) and Xinfeng Tang (tangxf@whut.edu.cn).

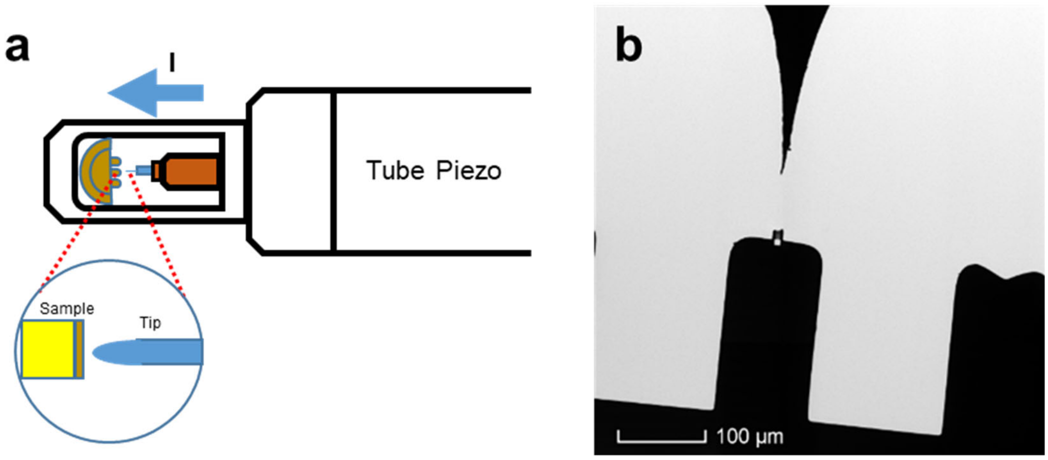


**Supplementary Fig. 1. Schematics of the in-situ TEM settings** with a scanning tunneling microscopy (STM)-like probe with a sharp tip (a); and a TEM image showing the STM probe along with the sample to be tested prepared by focused ion beam (FIB).

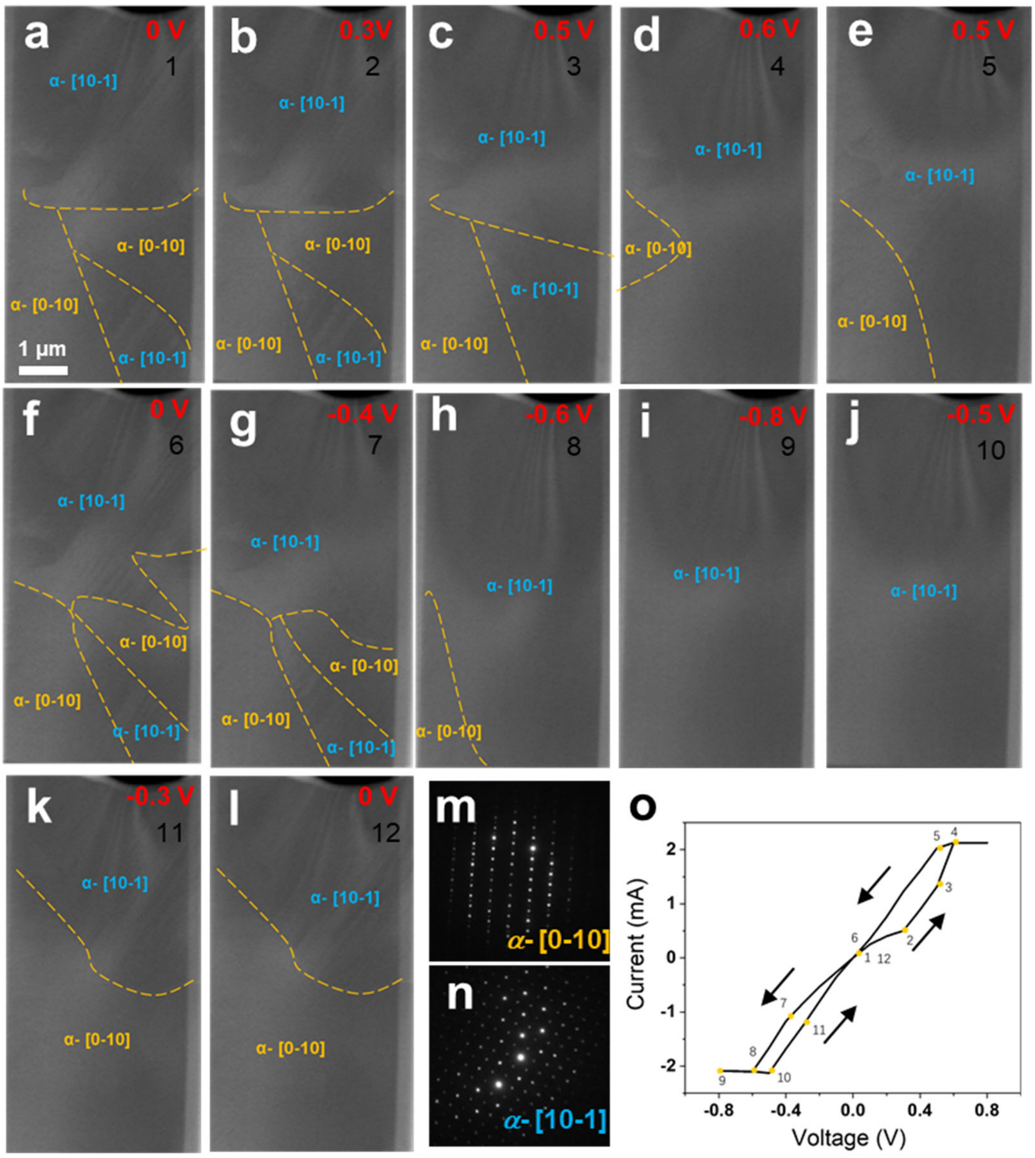


**Supplementary Fig. 2. Domain reconfiguration and corresponding electroresistance of *α*-$Cu_2Se$ under the fully cycled voltage.** (a-l) STEM images of the boundary (shown by a yellow dotted line) between the two domains, while the applied voltage is marked in each image. (m, n) Representative SAED patterns to identify the [0-10] and [10-1] domains, respectively. (o) Current-voltage characteristics of $Cu_2Se$ under the voltage cycle.

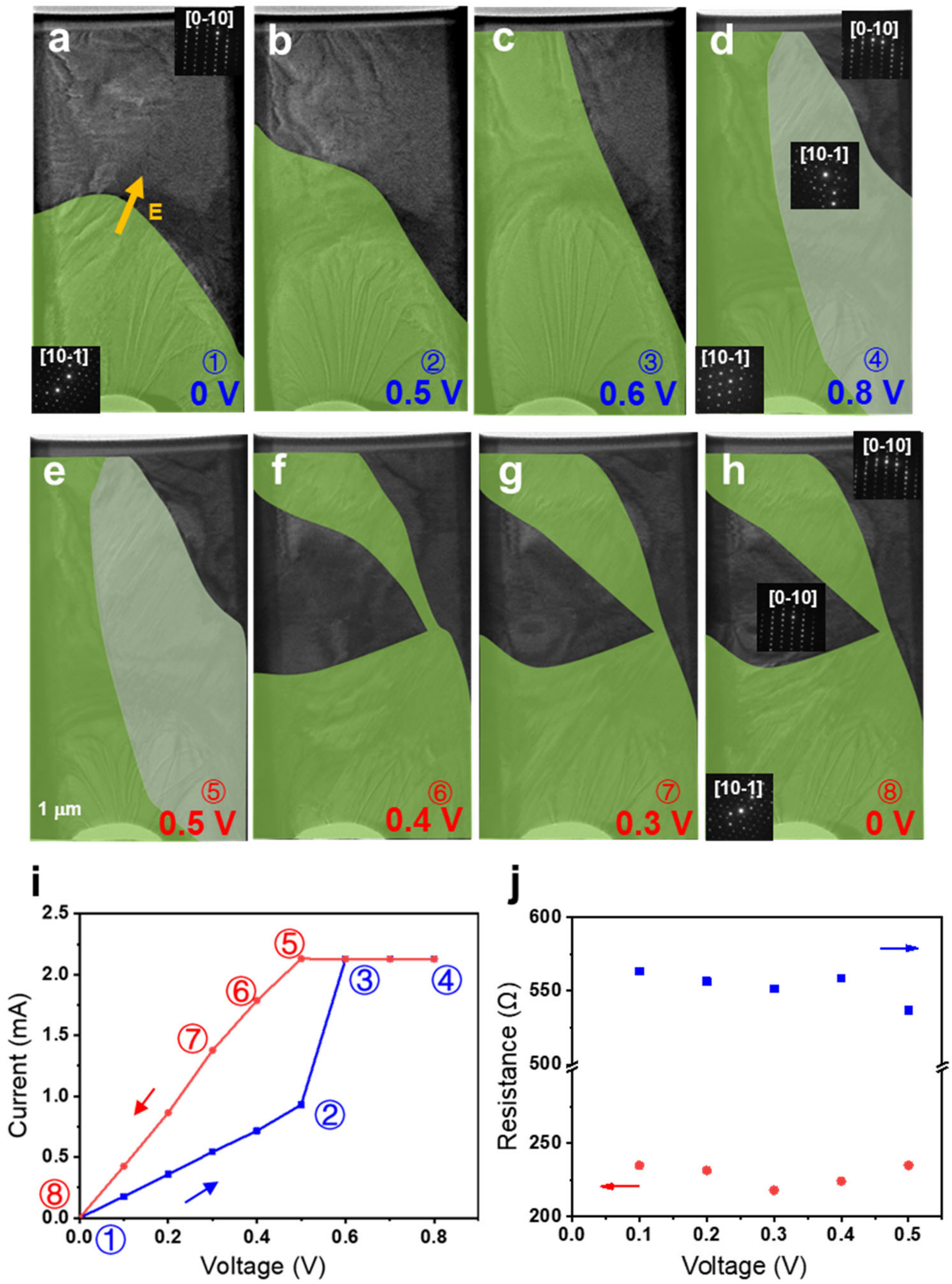


**Supplementary Fig. 3. Migration of the domain boundary of *α*-$Cu_2Se$ under the bias voltage.** (a-d) TEM images of the boundary between the two domains (the green area marks the [10-1] domain) during the progressively increasing bias from 0 V to 0.5 V to 0.6 V, and to 0.8 V, respectively. The insets show the selected area electron diffraction (SAED) of the corresponding areas. (e-h) TEM images of the boundary between the two domains during the decreasing bias from 0.5 V to 0.4 V to 0.3 V and to 0 V, respectively. The migration details can be found in the Videos S2-S3. (i) Current-voltage characteristics of $Cu_2Se$ under the applied voltage. (j) Resistive-voltage characteristics change of *α*-$Cu_2Se$ during the applied voltage.

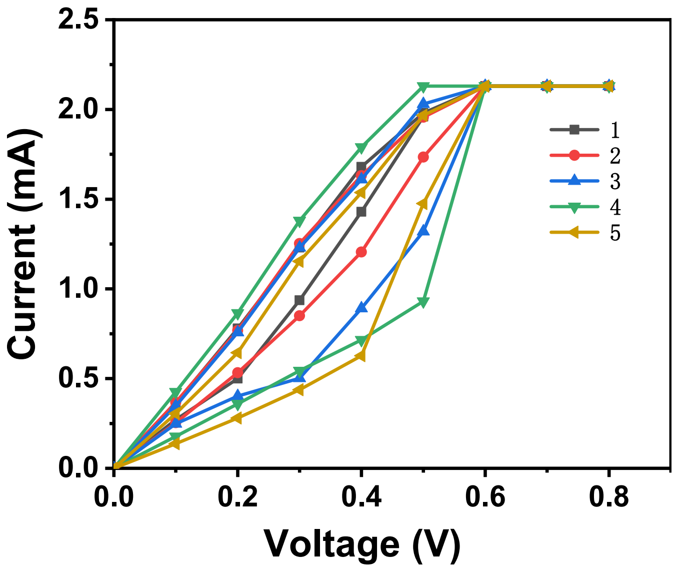


**Supplementary Fig. 4. Repeat cycles of electroresistance of $\alpha$-$Cu_2Se$ under the positive voltage in discontinuous scan mode.**

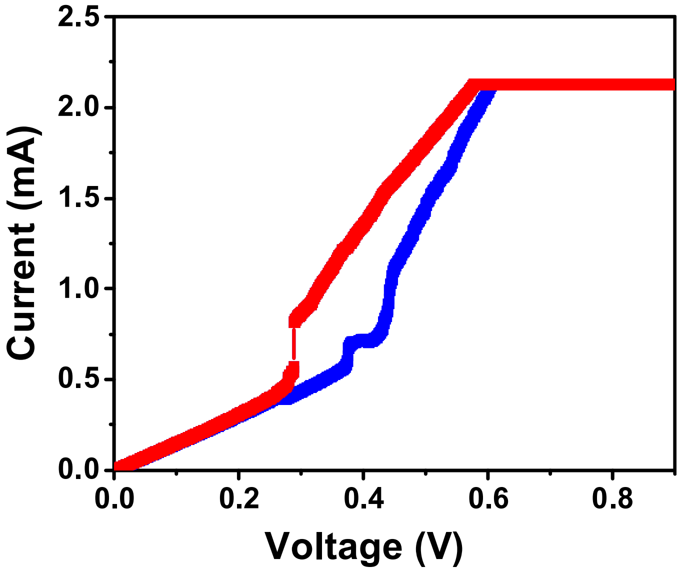


**Supplementary Fig. 5. Repeat cycles of electroresistance of *α*-$Cu_2Se$ under the positive voltage in continuous scan mode.** The migration details can be found in the Video S4.

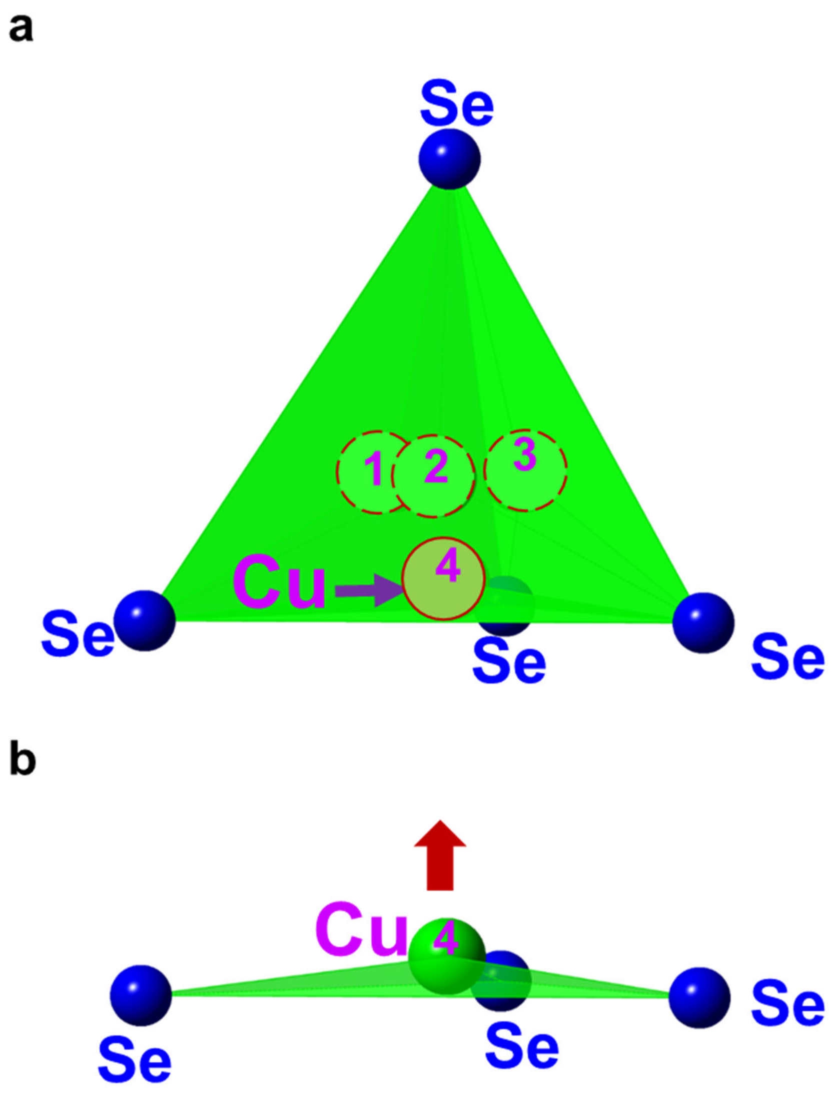


**Supplementary Fig. 6. Illustration of Cu-Se tetrahedron in $Cu_2Se$**. For clarity to show the ordering of the Cu-ions in $\alpha$-$Cu_2Se$, only the triangle (the closest to the Cu-ion) is shown in (b).

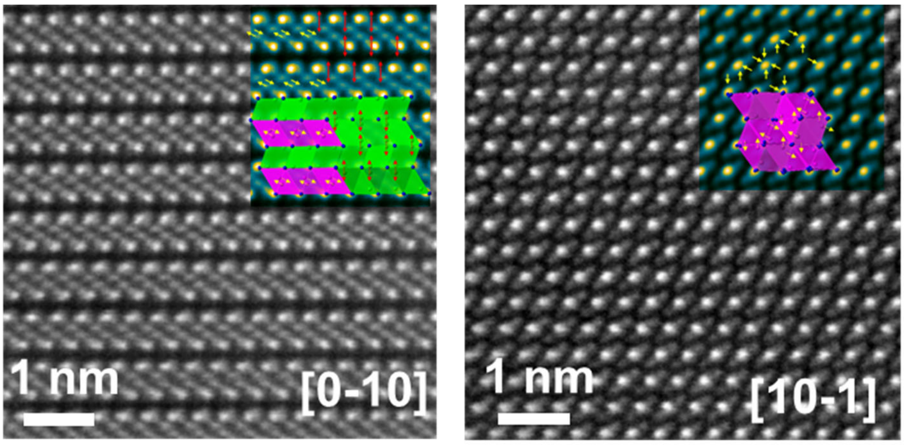


**Supplementary Fig. 7. The HAADF-STEM images of $\alpha$-$Cu_2Se$ along the [0-10] and [10-1] directions, respectively. The inset shown the polarization direction.**

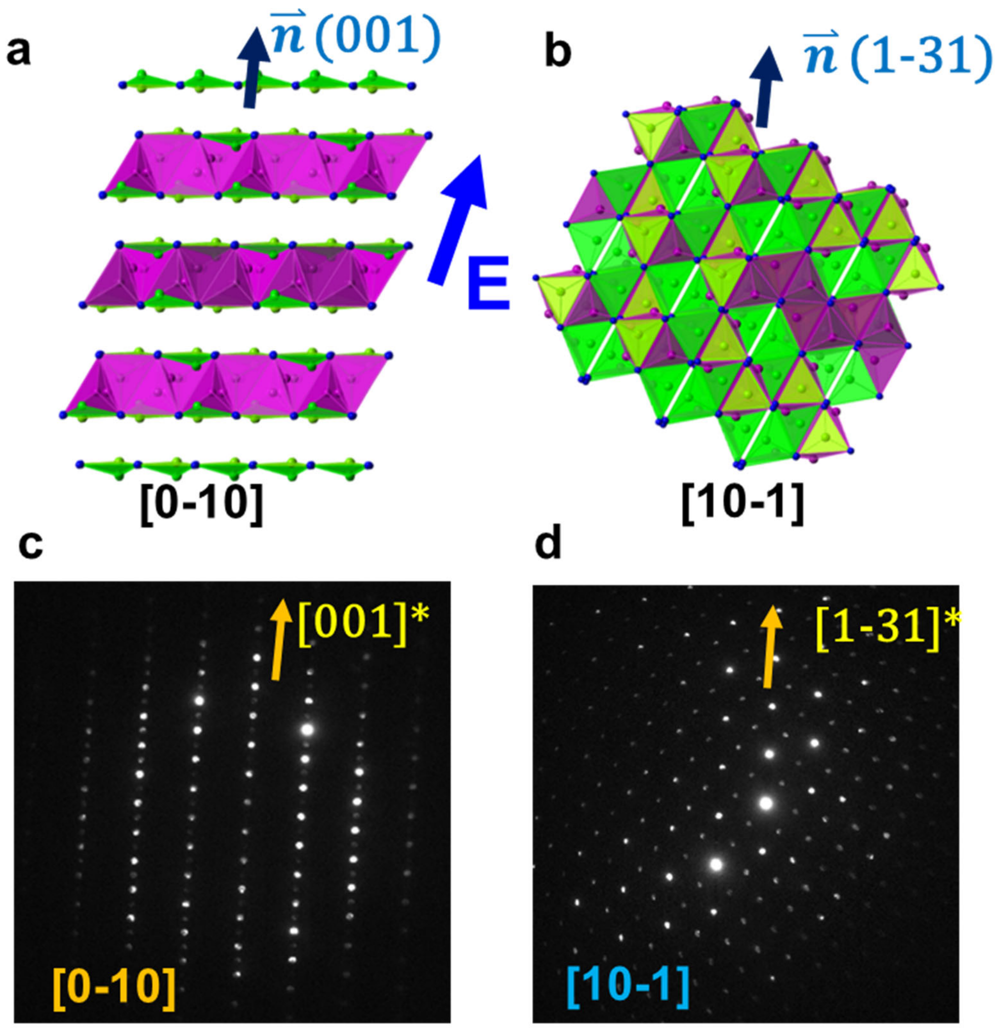


**Supplementary Fig. 8. Illustration of atomic structure of the two domains tuned by the applied electric field.** (a) The atomic structure of the [01-0] domain of $\alpha$-$Cu_2Se$ showing the layer-to-layer gaps. The [001] axis is also shown in the illustration. (b) The atomic structure of the [10-1] domain of $\alpha$-$Cu_2Se$ showing the [001] axis. Under applied electric field ($E$), the domain transformation is studied by SAED patterns, along (c) the [0-10] (c) and (d) the [10-1] directions, respectively.

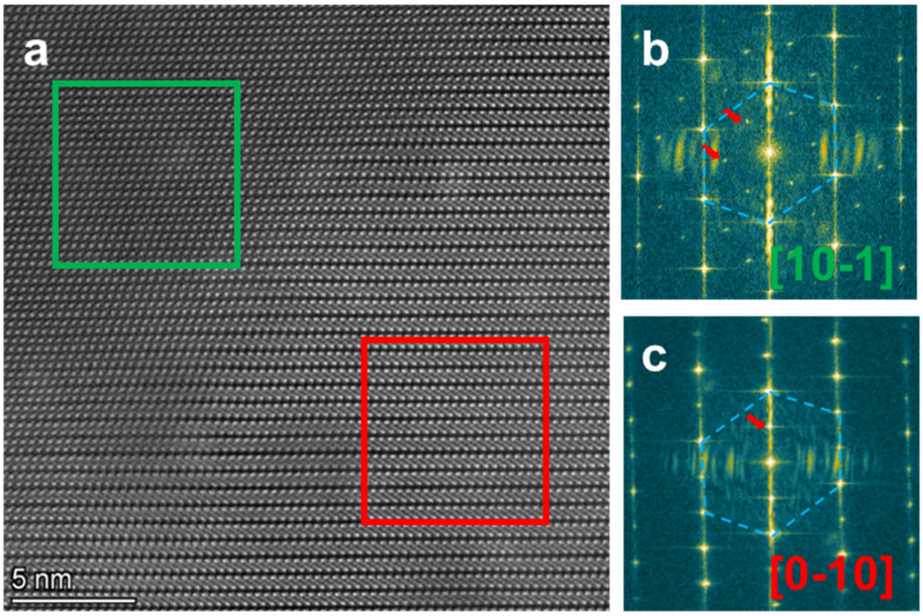


**Supplementary Fig. 9. Identification of the *α*-$Cu_2Se$ domain based on the STEM image through fast Fourier transform (FFT).** (a) The HAADF-STEM image of the domain interface, and the FFT of the area shown by a green rectangle (b) and a red rectangle (c). Extra spots can be found in (b), which is the proof of the [10-1] domain.

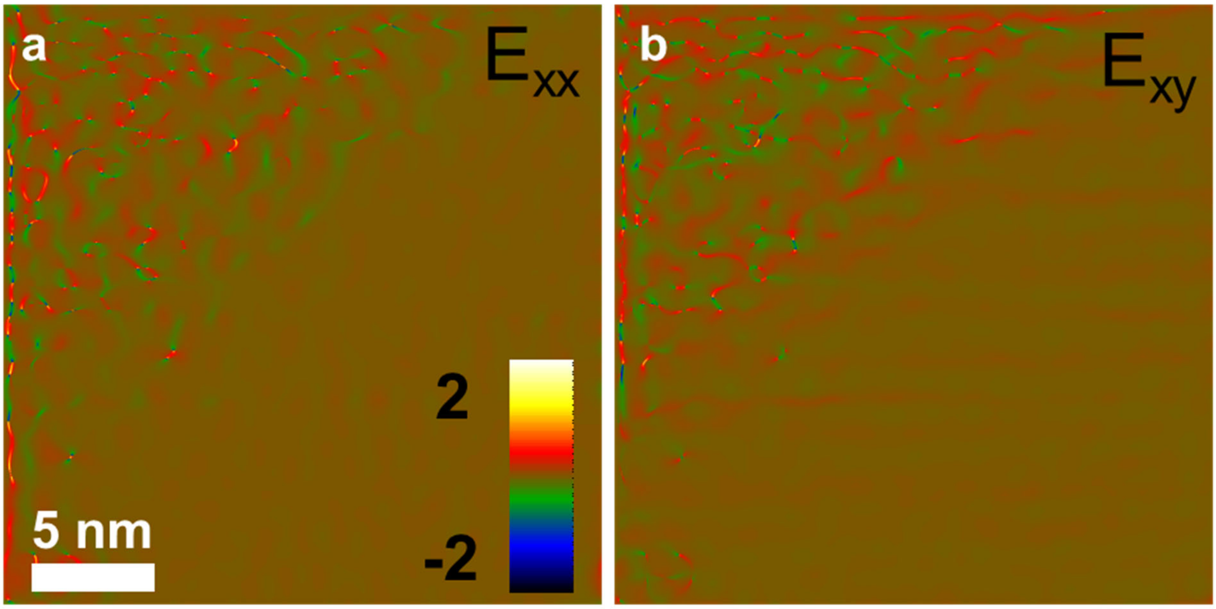


**Supplementary Fig. 10. The strain distribution of the domain interface calculated along two different directions.** Strain maps of the interface calculated by the GPA method along the *xx* (a) and *xy* (b) directions, respectively.

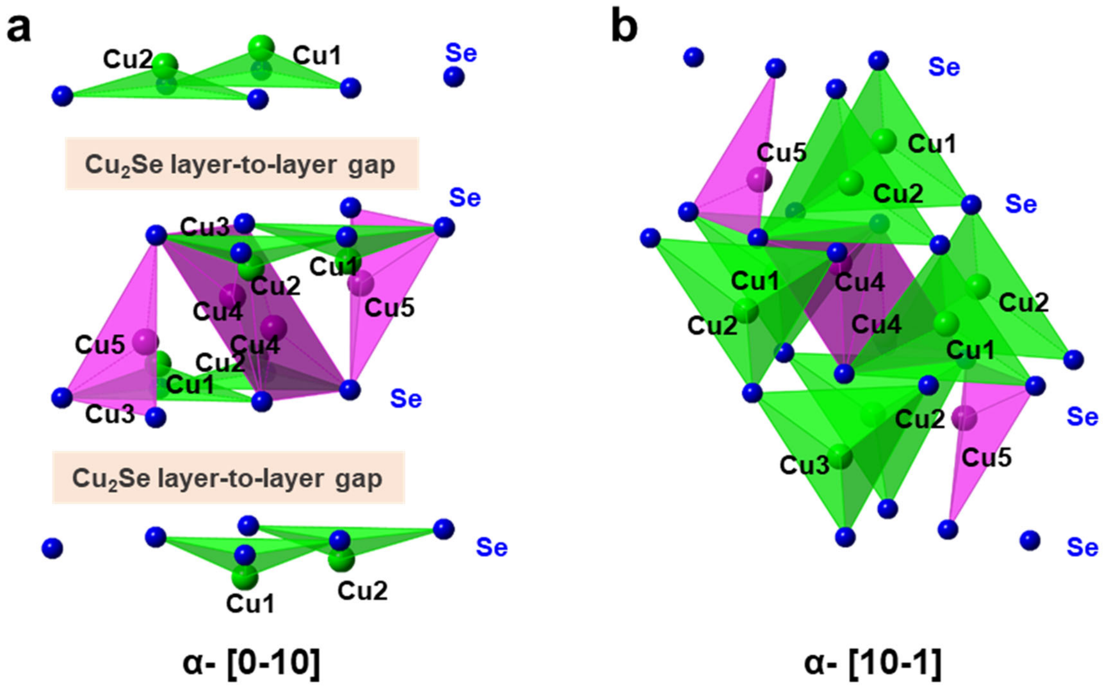


**Supplementary Fig. 11. Illustration of the Cu-ions hopping leading to the transformation from the [0-10] domain to the [10-1] domain in $\alpha$-$Cu_2Se$, while the Se-lattice remains almost unchanged.** (a) In the [0-10] domain, there exist layer-to-layer gaps where they are empty sites for Cu-ions. (b) In the [10-1] domain (transformed from the [0-10] domain triggered by the applied voltage), the layer-to-layer gaps have been filled by Cu-ions, demonstrating the hopping of the Cu-ions, such as Cu1 and Cu2.

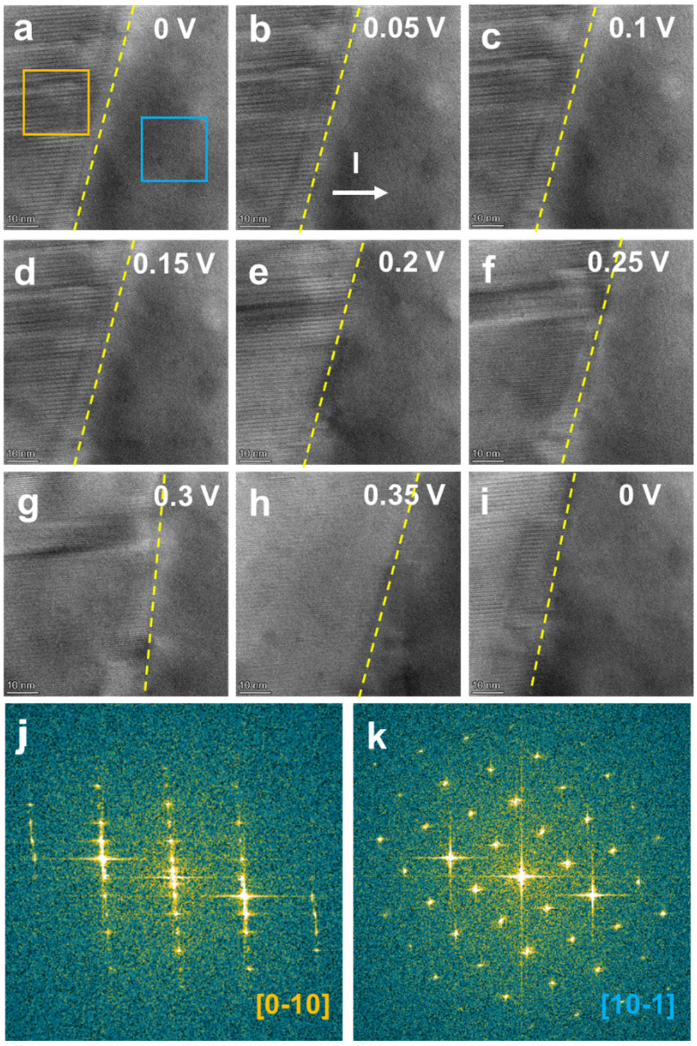


**Supplementary Fig. 12. Migration of the domain boundary of $\alpha$-$Cu_2Se$ under the bias voltage.** (a-d) HRTEM images of the boundary between the two domains at the different applied voltage. (j, k) The FFT of the area shown by a yellow rectangle and a blue rectangle in (a), respectively.

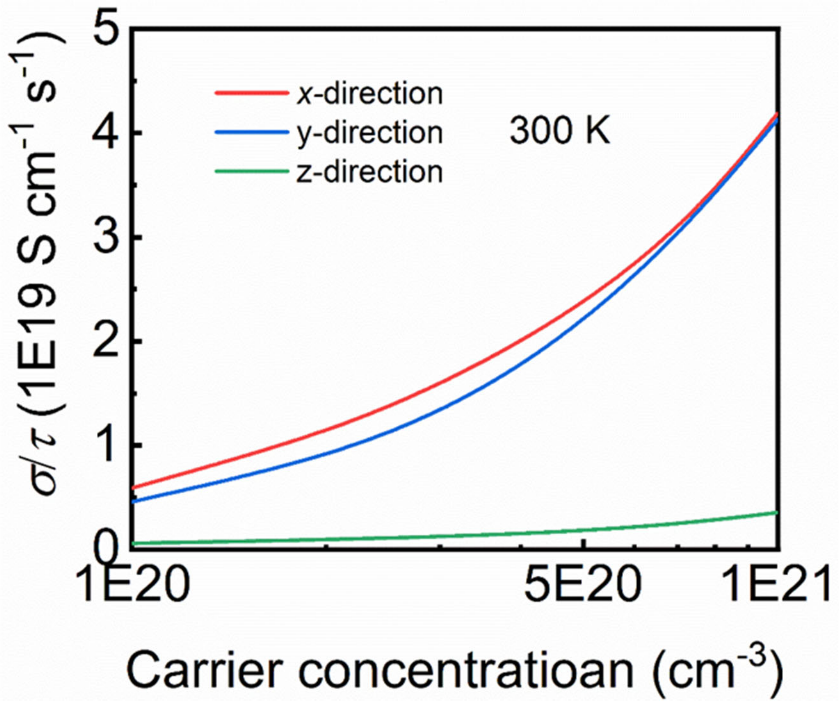


**Supplementary Fig. 13. The electrical conductivity at different directions of *α*-$Cu_2Se$ calculated by the density functional theory (DFT) as a function of the carrier concentration.**

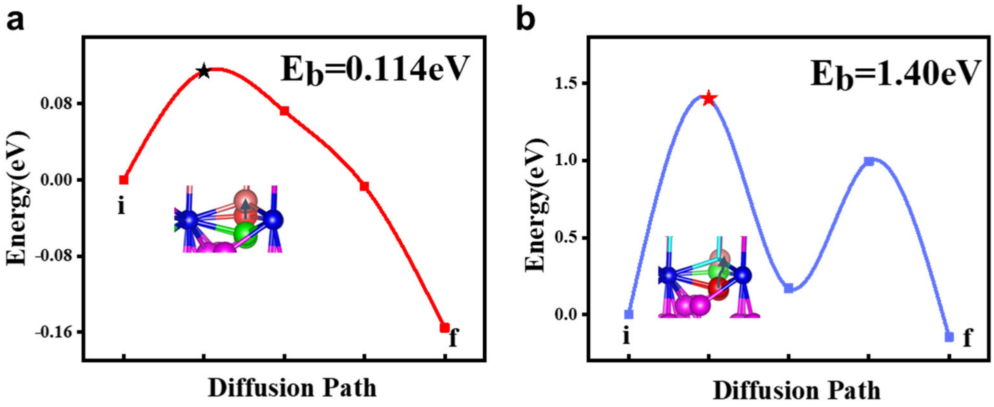


**Supplementary Fig. 14. The migration barrier of Cu-ions to the gap of *α*-$Cu_2Se$.** (a) and (b) show the migration barrier of the Cua from two different locations (shown in Figure 2) to the vacancy in the gap, respectively.

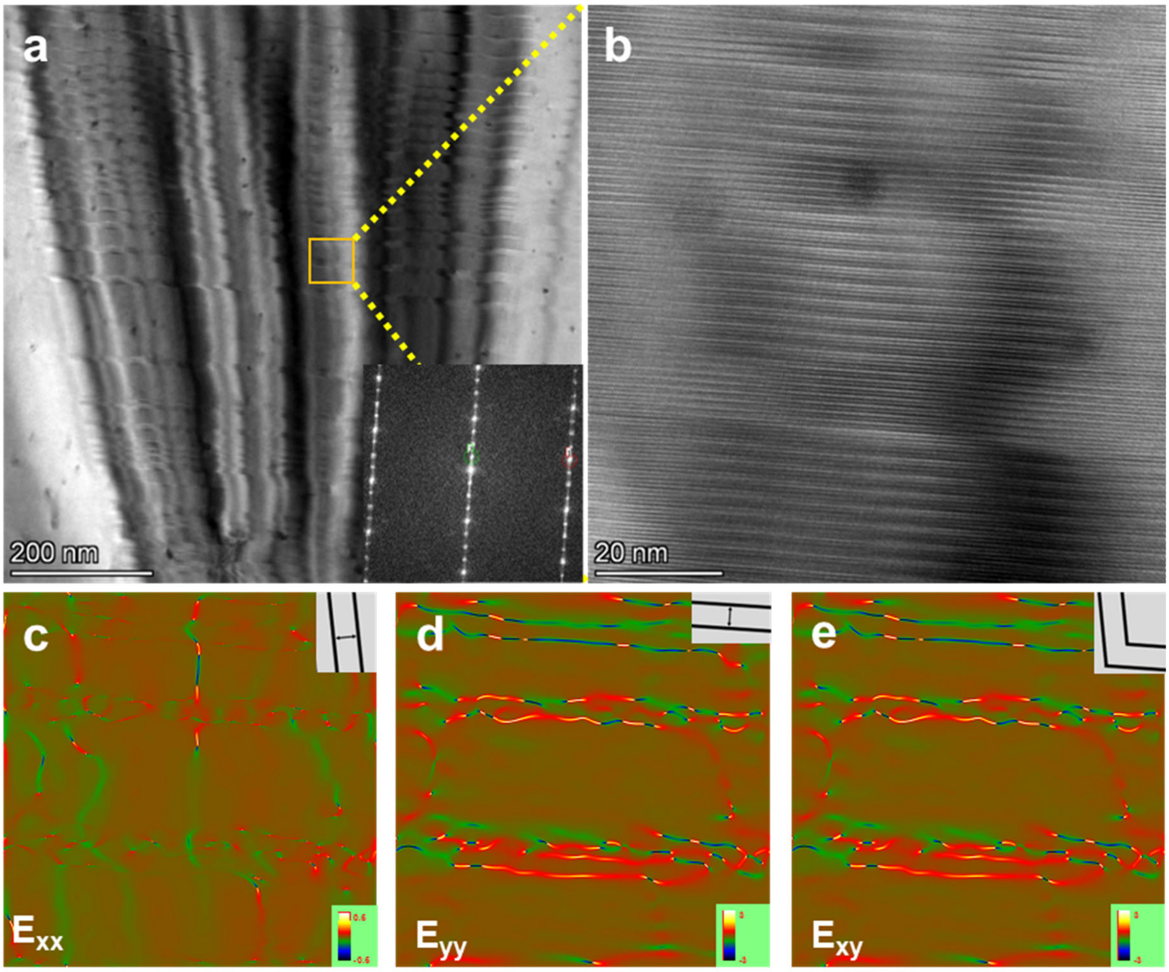


**Supplementary Fig. 15. Structural hysteresis in the voltage cycled $Cu_2Se$.** (a) A low-magnification TEM image of $Cu_2Se$ showing high density of planar defects. The inset is the corresponding fast Fourier transform (FFT) pattern, confirming it is the [0-10] domain. (b) An enlarged TEM image from the area indicated by the yellow box in (a), showing clearly the planar defects. (c-e) Normal strain maps along the *xx* and *yy* directions and shear strains along the *xy* direction, respectively, calculated from (b).

**Supplementary Movie 1. *In-situ* TEM of the electrically driven domain reconfiguration.** The migration of boundary of the $Cu_2Se$ domains v.s. the applied external voltage (×4 times faster than the real speed).

**Supplementary Movie 2. *In-situ* TEM of the boundary migration between the two domains under the increasing bias.** The migration of boundary of the $Cu_2Se$ domains v.s. the applied external voltage (×4 times faster than the real speed).

**Supplementary Movie 3. *In-situ* TEM of the boundary migration between the two domains under the decreasing bias.** The migration of boundary of the $Cu_2Se$ domains v.s. the applied external voltage (×4 times faster than the real speed).

**Supplementary Movie 4. *In-situ* TEM of the electrically driven domain reconfiguration under the positive voltage in continuous scan mode.** The migration of boundary of the $Cu_2Se$ domains v.s. the applied external voltage (×4 times faster than the real speed).

**Supplementary Movie 5. Migration of domain boundaries with the applied voltage is observed in the HRTEM.** The migration of boundary of the $Cu_2Se$ domains v.s. the applied external voltage (×4 times faster than the real speed).
